# The influence of corruption and governance in the delivery of frontline health care services in the public sector: a scoping review of current and future prospects in low and middle-income countries of south and south-east Asia

**DOI:** 10.1186/s12889-020-08975-0

**Published:** 2020-06-08

**Authors:** Nahitun Naher, Roksana Hoque, Muhammad Shaikh Hassan, Dina Balabanova, Alayne M. Adams, Syed Masud Ahmed

**Affiliations:** 1 BRAC James P. Grant BRAC School of Public Health, BRAC University, 5th Floor(Level-6), icddrb Building, 68 ShahidTajuddin Ahmed Sarani, Mohakhali, Dhaka, 1212 Bangladesh; 2 Department of Global Health and Development, London School of Hygiene and Tropical Medicine (LSHTM), Room TP 308, 15-17 Tavistock Place, London, WC1H 9SH UK; 3 Department of Family Medicine, Faculty of Medicine, McGill University, 5858 Cote des Neiges, Room 332, Montréal, Québec, H3S 1Z1 Canada

**Keywords:** Health-sector corruption, Good governance, Frontline health care services, Frontline health care providers, UHC, LMICs

## Abstract

**Background:**

The dynamic intersection of a pluralistic health system, large informal sector, and poor regulatory environment have provided conditions favourable for ‘corruption’ in the LMICs of south and south-east Asia region. ‘Corruption’ works to undermine the UHC goals of achieving equity, quality, and responsiveness including financial protection, especially while delivering frontline health care services. This scoping review examines current situation regarding health sector corruption at frontlines of service delivery in this region, related policy perspectives, and alternative strategies currently being tested to address this pervasive phenomenon.

**Methods:**

A scoping review following the Preferred Reporting Items for Systematic Reviews and Meta-Analysis (PRISMA) was conducted, using three search engines i.e., *PubMed, SCOPUS and Google Scholar*. A total of 15 articles and documents on corruption and 18 on governance were selected for analysis. A PRISMA extension for Scoping Reviews (PRISMA-ScR) checklist was filled-in to complete this report. Data were extracted using a pre-designed template and analysed by ‘mixed studies review’ method.

**Results:**

Common types of corruption like informal payments, bribery and absenteeism identified in the review have largely financial factors as the underlying cause. Poor salary and benefits, poor incentives and motivation, and poor governance have a damaging impact on health outcomes and the quality of health care services. These result in high out-of-pocket expenditure, erosion of trust in the system, and reduced service utilization. Implementing regulations remain constrained not only due to lack of institutional capacity but also political commitment. Lack of good governance encourage frontline health care providers to bend the rules of law and make centrally designed anti-corruption measures largely in-effective. Alternatively, a few bottom-up community-engaged interventions have been tested showing promising results. The challenge is to scale up the successful ones for measurable impact.

**Conclusions:**

Corruption and lack of good governance in these countries undermine the delivery of quality essential health care services in an equitable manner, make it costly for the poor and disadvantaged, and results in poor health outcomes. Traditional measures to combat corruption have largely been ineffective, necessitating the need for innovative thinking if UHC is to be achieved by 2030.

## Background

The goal of Universal Health Coverage (UHC) is that everyone needing health care can access quality services without financial hardship, and attains sustainable health outcomes [1]. To facilitate the journey towards UHC in low- and middle income- countries (LMICs), improving Primary Health Care (PHC) through health systems strengthening is essential [2]. However, ‘corruption’ contributes to undermine the UHC goals of equity, quality, and responsiveness of health systems and ultimately results in high, sometimes catastrophic, health care expenditures [3, 4]. Corruption, the ‘use, misuse or abuse’ of public office or resources for private gain [5, 6] may be actual or potential, financial, or even political [7]. It represents an abuse of trust and intentional violation of duty [8], and results in negative impacts on population health outcomes especially for the poor and the disadvantaged. For example, corruption (according to Transparency International’s corruption perception index) is estimated to be responsible for 140,000 annual deaths globally among under five children [9]. Higher levels of corruption (according to the same index) has been found to be associated with self-reported poor general health of both men and women, within all socio-economic groups across the lifecycle, in 20 African countries [10]. Also, corruption has beenincriminated as a cause in deaths from road traffic accident crashes, and deemed to be a necessary condition for effectively tackling road safety problems [11].

Corruption is a usual consequence of poor governance characterized by lack of transparency, weak accountability and inefficiency, and lack of citizen participation [12]. The importance of governance on the delivery of effective health care services cannot be overemphasized, even in situations where greater investments in the public health sector are occurring in tandem with economic growth [3]. ‘Good governance’ facilitates better allocation and efficient use of available resources including better and effective targeting of priority population groups for intervention [13, 14]. In a recent study based on national level panel data for ~ 148 countries, quality of government has been found to directly impact upon the infant and under-five child mortality, maternal mortality, and life expectancy [15]. The authors found that reducing corruption, one of the tenets of quality government, can significantly reduce infant and under-five child mortality.

The issue of corruption in the health sector is pervasive [16]. The particular susceptibility of the health sector to corruption arises from the asymmetry of information that characterizes the patient-provider relationship, the uncertainty that surrounds the illness experience, the multiplicity of actors and services involved in illness care, and the challenges of ensuring accountability in complex health systems [17]. Expensive hospital construction, high tech equipment and the increasing arsenal of medicines needed for treatment, combined with a powerful market of vendors and pharmaceutical companies present opportunities for corruption in the health sector. These opportunities increase when there is insufficient accountability for decisions or results in organisations. Organisational culture can further legitimize this behaviour by influencing individual attitudes and norms towards corruption and making it socially acceptable [18].

There is a rising trend of corruption in LMICs in the south and south-east Asian regions [19]. In these countries, the intersection between a pluralistic health system, a largely informal health market, and a poor regulatory regime have provided conditions favourable for corruption [20]. Besides, resource constraints in public sector resulting in poor salaries for the health care providers and inadequate supplies increase corruption such as informal payment and bribes [21, 22]. The conventional top-down approach of reward and punishment based on strict implementation of rules and regulations combined with various incentives to curb corruption (‘carrot and stick’ measure), has largely been unsuccessful in these countries [23]. This is partly due to the fact that health sector corruption is most often manifested at the level of patient encounter where providers (as ‘street-level bureaucrats’) have the freedom to shape the type and quality of care provided in the privacy of the doctor’s office [24].

Given the fact that traditional top-down approach of combating corruption through regulatory mechanisms and incentives have largely failed in LMICs, alternative, innovative approaches are needed that make the ‘cost of corruption’ outweigh its benefits [23, 25]. To identify bottom-up strategies that are perceived as ‘win-win’ for the stakeholders involved, an understanding of what is happening and why at the forefront of health care service delivery is necessary. Focusing on LMICs in the south/south-east Asian region, this scoping review documents and critically assesses relevant evidence on health sector corruption at the frontlines with the purpose of filling-in this knowledge gap. Study findings are expected to help policy makers and practitioners design innovative interventions that go beyond the traditional approaches to contain corruption.

## Methods

A scoping review following the Preferred Reporting Items for Systematic Reviews and Meta-Analysis (PRISMA) was conducted, using three search engines i.e., *PubMed, SCOPUS and Google Scholar*. A protocol was developed to guide the literature review process which specified study objectives, key research questions, inclusion/exclusion criteria, data sources (Table 1), and key search terms (Table 2). A PRISMA extension for Scoping Reviews (PRISMA-ScR) checklist was filled-in to complete this report (Additional File 1). Data were extracted using a pre-designed template and analysed by the ‘mixed studies review’ method [26].

**Table 1:** Literature review protocol

**Objectives**	To understand current scenario and policy perspective in selected countries of the south and south-east Asia region to address irregularities and informal practices in the public sector frontline (PHC) health care facilities	
	To identify different types of irregularities and informal practices perpetrated by the frontline health care service provider in countries of south and south-east Asia	
	To explore different innovative approaches practiced in this region to address irregularities and informal practices.	
**Research Questions**	What are the different types of irregular and informal practices perpetrated by the frontline health care service providers in the public sector?	
	What motivates them to engage in corrupt practices?	
	What are the conditions that incentivize corrupt behavior among them?	
	Which anti-corruption initiatives promote transparency and accountability among public sector health care providers?	
	How does the legal and regulatory framework promote or constrain corrupt practices by these providers?	
**Search Strategy**	Inclusion Criteria	Peer-reviewed, full-text articles, all methods, all design, written in English
		Policy data involved with health sector corruption and governance from central to community
		Grey materials (published and/or unpublished)
	Exclusion criteria	Corruption related literature not with a reference or focus on health sector
	Time frame	January 2007 to August 2017
	Selected south and south-east Asian LMICs	Bangladesh, Bhutan, India, Indonesia, Nepal, Sri Lanka, Myanmar, Vietnam, Philippines
**Data source**	Electronic database	PubMed, SCOPUS and Google Scholar
	Grey literature	National Bangla & English newspaper articles; Health related blog in English and Bangla; Books/ Monograph/ dissertation/ conference paper/conference proceedings
	Organizational website	The World Bank, Anti-corruption Resource Centre, Transparency International Bangladesh, Center for Global Development, CHR Michelson Institute, WHO, Ministry of Health & Family Welfare (MoHFW), Director General of Health Services (DGHS), Director General of Family Planning (DGFP),
	Journal’s website	PLoS ONE, BioMed Central, Lancet, Health Policy and Planning etc.

**Table 2:** Key terms used for searching electronic databases

Corruption (combined by ‘OR’) (a)	Governance/accountability (combined by ‘OR’) (b)	Health sector (combined by ‘OR’) (c)	Geographic location (combined by ‘OR’) (d)
Corrupt	Governance	Hospital	Bangladesh
Bribery	Good governance	Health care center	Bhutan
Dishonesty	Accountability	Health facilities	India
Anti-corrupt	Community accountability	Health service	Indonesia
Tackle corrupt		Health worker	Myanmar
Combat corrupt Anti-corrupt strategy		Frontline health care provider	Nepal
Rent seeking behavior		Manager	Sri Lanka
Informal payment		Administrator	Philippines
Informal practice		Service provider	Vietnam
Illegal practice			LMICs
Elicit practice			LICs
Speed up money			

Note: a,b,c,d groups were combined with Boolean operator ‘AND’

### Search strategy

**Fig. 1:**
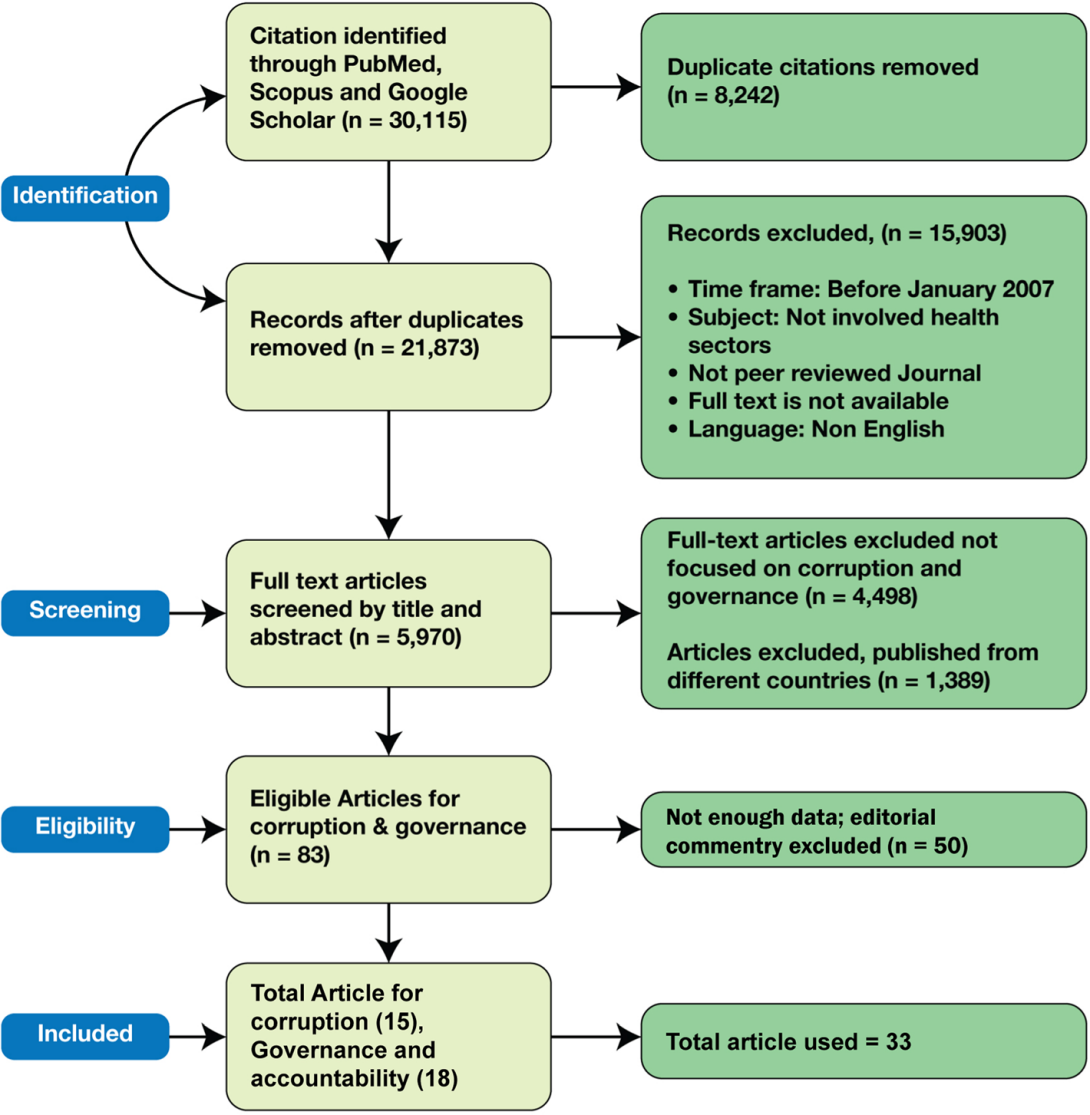
PRISMA Flow Diagram

We searched information from published articles and documents as shown in the PRISMA flow diagram (Fig. 1). The search strategy and terms were developed collaboratively with our study partners. Search strategy focused on following key words: corruption, informal payment, rent-seeking behaviors, bribery, anti-corruption strategy and/or behavior, governance/ good governance and/or accountability in frontline health workers/managers, service providers of health facilities/system, hospitals, etc. The literature search was conducted during Sept. – Oct. 2017 and the full search strategy is available on request from the authors. Citations for journal articles were managed using EndNote X7.7.1 software.

### Operational definitions

For our purpose, the key concepts of the study are defined as follows (10): i) Corruption: the abuse of entrusted power for private gain (actual or potential, to be realized in the future), alternatively described as “irregularities and informal practices and/or rent-seeking;” also encompasses concepts of transparency (honesty and openness) and accountability (‘the extent to which people, groups and institutions are able to hold government and other power holders responsible for their actions’), the two pillars of ‘good governance’; ii) Informal payment: payments made to health providers for services supposed to be provided free of charge or payments made that are greater than official fees; (alternative terms used: ‘under the counter/table payment,’ ‘envelop payment,’ ‘unofficial fees’ etc.), payments made outside official channels, payments over and above fixed co-payments, and additional payments for entitled services [27] iii) Bribes: cash or kind exchanged with the health service providers/officials against an official action (e.g., posting and transfer to health facilities of choice); iv) Absenteeism: stealing time by not coming to work or doing private practice during working hours [28]; v) Frontline health care services and frontline health care workers: Essential health care services delivered by the doctors, nurses, paramedics, CHWs and lab and pharmacy technicians at the public sector sub-district (*upazila*) health facilities and below (the formal level of PHC); vi) Regulatory regime: arrangements of steering and control mechanisms that influence the operation of health systems e.g., statutory measures for controlling the practice of medical profession or health facilities, or marketing of medicines and medical devices etc. vii) Decentralisation: a socio-political process that transfers authority and responsibility in planning, management and decision-making from central government to local government bodies.

### Data extraction and synthesis

Two researchers from the study team identified the articles independently from electronic databases, grey literature, and manual search. After excluding duplications, screening titles and abstracts, and applying eligibility criteria, a total of 36 articles and documents were included for data extraction and analysis (Fig. 1). A pre-designed template was used for extraction of data from the selected articles and documents. The two key themes of the study viz. corruption and governance, were subsequently disaggregated into sub-themes for further granularity: corruption (types and forms, practices, opportunities and incentives, impact) and governance (transparency, accountability, laws and regulations, citizen engagement) (Table 3). We used a ‘mixed studies review’ method to synthesize evidence from quantitative, qualitative and mixed methods studies, which is particularly effective when analyzing complex public health interventions [26]. Any confusion with categorization or data interpretation was resolved by discussion among research group members under the supervision of the PI.

**Table 3:** Themes and sub-themes for framework analysis

Themes	Sub-themes	Example of codes used
**Corruption**	• Different forms of corruption • Corruption practices • Conditions that incentivize corruption • Impact of corruption and organizations that promote corruption	• Bribery, informal payment, absenteeism, medicine irregularities • Administrative practice, pharmaceuticals, diagnostic practice, service delivery related practice • Medical education, career prospect, informal payment • Use of resources, access & utilization of services,
**Governance**	• Transparency and accountability • Citizen participation • Anti-corruption initiatives • Laws and regulations of anti-corruption	• Community ownership, patient welfare society, hospital management committee, policies to combat corruption

## Results

Out of the 33 documents included for analysis, two were systematic reviews, nine were reviews,and 16 primary research papers. Two blogs and four reports identified through manual search were also included. Corruption related documents dealt with the types, causes and consequences of corruption while governance-related documents mostly focused on decentralization, e-governance, and the concept of ‘good governance’. Only six of the documents reviewed dealt with the issue of accountability as an essential element of good governance. Interestingly, literature fulfilling the search criteria for selected countries were limited in number, and the majority of relevant documents were from India. Given the wide prevalence of corruption in these countries (Bangladesh, Bhutan, India, Indonesia, Nepal, Sri Lanka, Myanmar, Vietnam, Philippines) [19], the relatively small number of published materials in these countries suggest a substantial evidence gap in this area which hamper the design of informed interventions for its containment. To further enhance the depth, breadth and richness of the study findings, researchers developed and analysed relevant sub-themes and then grouped these into two key study themes namely, Corruption (Table 4) and Governance (Table 5), and are presented below.

**Table 4:** Summary of studies exploring different dimensions of ‘corruption’ (*n* = 15)

Author; year [ref]	Type of study	Settings	Themes/sub-themes	Findings
Mannan MA; 2013 [29]	Survey using both quantitative and qualitative methods	Nationally representative sample from seven divisions included 14 district and 28 sub-district hospitals and 28 union hospitals in Bangladesh	Informal payment/hospital admission	• Informal payments to facility staff (mainly non-technical) hastened the process of getting admission in a public health facility • About 1/3rd of inpatients in district hospital and 1/5th in sub-district hospitals made an extra payment for getting admission; 8% of them made extra-payments at least three times and occurred more for the poor as they had no connection to or recommendation from influential people • In FGDs, 50% of the participants said that they made informal payments because they feared that without these extra payments’ they would either receive no treatment at all or would be subjected to neglect/slow treatment.
Paredes-Solís et al.; 2011 [30]	Mixed methods	South Asia (and Africa and Europe)	Informal payment/ impact	• It causes disproportionate financial burden to the poor households due to paying for supposedly free services and non-availability of medicines in hospitals • **Impact**: Thus, dissatisfaction with services ultimately results in gradual decrease in the use of government services over time • **Social audits** may be an important tool to identify corruption at service delivery points such as informal payments
Lewis M; 2007 [31]	Review	LMICs	Informal payment/ causes and impact	• Informal payment is quite common in south/south-east Asia e.g., Pakistan (96%), Bangladesh (60%), India (24%), Cambodia (55%), Vietnam (81%). • **Causes:** Low pay, irregular salary payments, poor governance and management • **Impact:** Increases inequity in access to health care services.
Stepurko et al.; 2010 [32]	A systematic review of research methods and instruments	39 countries including LMICs	Informal payment	• Methodologically, self-administered questionnaires were found to be suitable in a face-to-fact interview for collecting information of sensitive nature such as informal payment
Matsushima M & Yamada H; 2016 [33]	Cross-sectional study; household survey	Ho Chi Minh city and Hanoi, Vietnam	Bribery/ causes and impact	• Bribery is common in instances such as enrolling members in health insurance schemes, provision of certain services, bypassing queue to reduce waiting time, transfer and posting of choice, taking unlawful leave etc. • Bribery is negatively correlated with health outcomes and insurance coverage • **Causes:** Hospital directors’ autonomy regarding staff hire and fire, salary and perks, transfer and promotion etc. health care workers sell services at higher prices • **Impact:** Increased price of services
Nguyen VH; 2008 [34]	Survey Data	36,000 Households in Vietnam	Bribery	• Social interactions in the form of advice on choice of a hospital for a particular service leads to an increase in the propensity to bribe and the amount of bribe.
Azfar O &Gurgu, T; 2005 [35]	Quantitative Survey	1100 households and 160 health workers from 80 municipalities in 19 provinces of Philippines	Bribery/ impact	• Bribery reduces immunization coverage, delays newborn vaccination, increases waiting time and discourages public health series • **Impact:** Bribe increase the cost to consumers which reduces demand for services, reduces Government resources allocated to service delivery, affects health outcome in the rural areas • Affects of corruption vary by region (rural or urban), and also, affects the poor disproportionately
Abdallah W et al.; 2015 [36]	IZA discussion paper	12,240 Households in Bangladesh	Bribery/ causes and impact	• 41% patients pay illegal consultation fees in public sector health facilities; patients who were living further away from the health facilities, were paying more bribe • **Causes:** Low payment of health care workers and lack of incentives • **Impact:** Patients have to wait for a long time to get the service, the Quality of Care of services provided was low for non-bribing patients.
Azad A; 2014 [37]	Blog (Bangladesh health sector)	Survey from 28 health institutions	Bribery/impact	• Bribe was paid during recruitment of ad hoc doctors, 3rd – 4th class employees, transfer posting from Upazila to capital etc. • Doctors earned money from commission agreements with diagnostic centers thereby driving up costs of care etc.
Nanjunda;2014 [28]	A cross-sectional survey, informal interview, and participant observation	30 selected Community Health Centers (CHCs) in South Karnataka	Absenteeism/ causes and impact	• Unauthorized absence is more prevalent among doctors (27%), followed by lab technicians (17%) and female nurses (13%); mostly in the afternoon and around weekends (17%); 76% of doctors were engaged in private practices or running their clinics • **Causes:** More senior health workers having good relationship with higher authority leads absenteeism in the government facilities, lack of sufficient number of staff and residential facilities for doctors and nurses
Ramadhan and Santoso; 2015 [38]	Quantitative survey	9 community health centers in Benkulu city, Indonesia	Absenteeism/ causes and impact	• Unauthorized absence is found to be 26.5% among Doctors, followed by 23% among midwives and 23% among para-medics • **Causes:** Most of the doctors are living in city areas, and they leave facilities early, especially on the last working day. • **Impact:** the poor and disadvantaged cannot receive appropriate and timely treatment
Lewis, M; 2006 [39]	CGD Brief Report	LMICs	Absenteeism/ causes and impact	• Absenteeism rate among health workers was 19% (Papua New Guinea) to 75% (Bangladesh) • **Causes:** Low wages and irregular payments of wages forced workers to indulge in additional income-earning activities which causes absenteeism in the government health facilities • **Impact:** interference in service delivery, both quantitatively and qualitatively
McDevitt et al.; 2015 [40]	Anti-corruption Resource Centre Document	Bangladesh	Absenteeism	• Absenteeism rates in primary health care centres in Bangladesh to be as high as 35% • The regulatory framework for monitoring health service delivery is weak, with 45 separate laws related to various aspects of health
Knox C; 2009 [41]	Quantitative survey	5000 household survey in 52 districts govt. facilities in Bangladesh	Absenteeism/Negligence	• 42% of the patients encountered corruption while accessing services and 43% faced negligence by professionals, disproportionally affecting the poorest disproportionately • **Impact:** Lack of trust in health care providers and ultimately lesser use of services by the poor and the disadvantaged
Hipgrave and Hort; 2014 [42]	Review	LMICs of south/south-east Asia	Absenteeism/dual practice	• ‘Dual practice’ by health professionals is quite common in south/south-east Asia (e.g., in Bangladesh it is cited to be around 80% while in Indonesia from 70 to 80%) • Poor regulation of dual practice encourages absenteeism and negatively affects access, quality and equity of services provided

**Table 5:** Summary of studies exploring different dimensions of ‘Governance’ (*n* = 18)

Author, year (country)	Type of study	Settings	Themes/sub-themes covered	Findings
Rose J et al., 2014 [43]	Systematic review	Bangladesh health sector since 2000	Transparency /accountability: public sector	• Some pertinent governance issues included corruption of inventory management, high rate of absenteeism of health care service providers and problems of human resource management • There is limited transparency in government regarding basic data e.g., procurement procedures at the national level • public doctors unnecessarily refer patients to private clinics or practices.
Nurunnabi M and Islam SK, 2011 [44]	Survey and Secondary research	533 patients from 45 hospitals in Dhaka city of Bangladesh were surveyed using questionnaire	Transparency/ accountability: private sector	• In the private health care sector, four factors were found to be significantly associated with accountability, in order of influence: professionals, administration and management, legal enforcement and ethics, and government
Ghimire, J et al.; 2013 [45]	Qualitative study	90 health facilities in Siraha, Bardiya and Doti districts in Nepal	Transparency /accountability: display of information	• Only 49 (54%) of the health facilities have properly displayed signboard, 42 (47%) citizen charter, 36 (40%) free health services and Information on Aama program in 25 (28%) health facilities. • 72 out of 90 health facilities have not displayed social audit reports, and 80 (89%) of the health facilities have not maintained complaint box.
Dieleman, M et al.; 2011 [46]	Review of case studies	A literature review of HRH management in LMICs; case studies identified through Scopus, PubMed, Embase	Transparency /accountability: managing HRH	• The review covered four dimensions of governance: performance, equity and equality, partnership, and oversight • In ‘oversight,’ local-level corruption affects accountability and local-level trust in governance • Experiences with accountability mechanisms for HRH policy development and implementation were lacking.
Kamal S et al.; 2014 [47]	Qualitative study using In-depth interviews, KII group discussion and secondary data	Public and private health care institutions in Bangladesh	Transparency /accountability: HRH	• 20% of total sanctioned post is vacant. Doctors do not have to be accountable for not attending the office on time. • The procedures of promotion, selection grade, and regularizing ‘In Charge’ positions are quite lengthy in the health sector. • Political pressures and influences are very prevalent when medical certificates on death and injuries are badly needed for filing police cases.
Garimella, S and Sheikh, K; 2016 [48]	Case studies on posting and transfer	Primary health care center in Tamil Nadu, India	Transparency /accountability: HRH	• Posting and transfer emerge as a complex phenomenon, shaped partially by the laws of the state and partially as a parallel system of norms and incentives requiring consideration and coordination of the interests of different groups. • Beyond a functional perspective of PT, it also reflects justice and fairness as it plays out in the health system.
Cleary, S et al.; 2013 [49]	Descriptive literature review	Review of PubMed literature in LMICs	Accountability: citizen participation	• Bureaucratic accountability mechanisms often constrain the functioning of external accountability mechanisms. • Citizen participation: community members are behaving like “watchdogs;” external supervision by community people can play a role for functioning external accountability mechanism.
Papp, S et al.; 2013 [50]	Case study	Civil society organizations in Orissa, India	Accountability: citizen participation	• Public hearings as a social accountability tool facilitate: (1) demand generation for better services, (2) leveraging intermediaries to legitimize demands of poor and marginalized women, and (3) sensitizing leaders and health care providers to women’s needs • The process involves raising critical consciousness among marginalized women and giving space to voice their concerns and demands to people in power and also, receptivity of the latter to hear their grievances and act on these
Roalkvam, S; 2014 [51]	Review	India	Accountability: citizen participation	• Rights of citizens are not solely contingent upon the existence of legally guaranteed rights but also significantly on the social conditions that make their effective exercise possible.
Lodenstein, E et al.; 2017 [52]	Review	37 social accountability initiatives in LMICs identified through a literature search	Accountability: citizen participation	• Perspectives of providers to citizen’s expectations and demands for better health care is essential for improving the quality of primary health care in different settings • Providers’ ‘receptivity’ to such demands expectations and their ‘relation’ to citizens for tapping personal and professional support for improved responsiveness can be understood and acted upon following a Context-Mechanism-Outcome theory of change
Islam MS and Ullah MW; 2009 [53]	Mixed method using case study approach	Muradnagarupazila health complex, Comilla, Bangladesh	Accountability: citizen participation	• Peoples’ have no involvement in decision making process in the health sector • Lack of proper economic management is hampering the participation of people in health services.
Regmi, K et al.; 2010 [54]	Review	Medline, PubMed, Embase, CINAHL, DARE literature review from Nepal	Decentralization	• Decentralization of health sector implies increased accessibility of the public to health services by increasing transparency and accountability • The restructuring of the district health care services from decentralization was considered the highest achievement for meeting the needs of the local community. • Decentralization is diverting the attention of the public away from central bureaucratic rules and gained popularity by installing local governments at the lower level.
Panda, B, and Thakur, H; 2016 [55]	Review	Focused literature review from India using PubMed and Google Scholar	Decentralization	• For exploring effects of decentralization, one needs to examine and assess the role and functions of local decision-making institutions and results thereof at institutions, systems, and individual levels • Decentralization of local self-governance in public health sector has multiple dimensions in conceptualization, measurement complexities, and byproducts for consideration.
Gurung, G, and Tuladhar, S; 2013 (Nepal) [56]	Quantitative study	28 districts	Decentralization	• Local health facility management committee ensured community engagement, mobilization of local resources, improved responsiveness and accountability to the community, and provision of inclusive health services • Availability of technical staff, supervision, and monitoring, and display of citizen charter improved accountability.
Rauniyar G et al., 2013 [57]	Performance Evaluation report	Indonesia; evaluation of ADB funded decentralized health services project	Decentralization	• Improved access to health care services, especially in remote and rural areas; the poor benefitted the most • Service delivery constrained by limited funding with a major proportion spent on administration • Continuous monitoring is essential for identifying current trends of decentralization
Millington KA and Bhardwaj M; 2017 [58]	Report	LMICs including India, Bangladesh	Good governance	• To address corruption in pharmaceutical procurement, drug pricing transparency is mandatory for good governance through drug pricing information from a govt. online database.
Roncarati, M; 2010 [59]	Review of examples	LMICs	Good governance	• Weak institutional capacities hinder good governance and better health outcome. • Generate awareness among stakeholders, supportive institutional structures, incentives and payment schemes enhance good governance
Huss, R et al.; 2010 (India) [60]	Qualitative study/ 44 semi-structured interviews	Government hospitals in urban and rural areas of Karnataka;	Good governance	• Good governance is the responsibility of all citizens for a responsive and inclusive health systems with fair outcomes • To combat corruption effectively, committed and powerful leadership, adequate resource and capacity to investigate senior government officials, and institutional reforms are needed • Concerted efforts from political and justice systems, media and awareness-building among the population is essential to succeed in anti-corruption actions

### ‘Corruption’

In our review, a long list of irregularities and rule-breaking or rule-bending practices (i.e., ‘corruption’) were identified in the context of health care service delivery transactions including informal payments, bribery and absenteeism (Table 4).

#### Informal payment

In the reviewed literature, examples of informal payments include payments made at different stages of service use in the public health facilities ranging from availing a trolley for patient transport to getting admission and securing a hospital bed to availing subsidized medicines from the hospital. Even if admitted, patients and attendants were afraid that without extra payment (to mainly non-technical staff), they would either receive no treatment at all or would be subjected to neglect/slow treatment, as they had no connection to or recommendation from, influential people [29]. Furthermore, financial barriers and patient frustration arising from petty corruption may work to hinder access and utilization of public sector facilities [30]. For example, only 24% of households in Pakistan visited Government Public Health facilities in 2004, while 45% consulted private medical practitioners. Similarly, in Bangladesh, only 10% of households visited government health care facilities, while health care-seeking from nongovernment organization (NGO) clinics increased from 30 to 49% of household in 2003. For those who do avail services, many feel trapped and forced to make these payments. The literature identifies social audits as a potentially effective method for revealing petty corruption at service delivery points including the practice of informal payments [30]. However, in some resource-poor settings such as Pakistan, Bangladesh, India, Cambodia and Vietnam, informal payments are viewed as a necessary source of financing health care and thus are rationalized and sustained within individual and organizational norms and practices [31]. Given the complexity and sensitivity of the practice, the literature recommends that information on informal payment is best elicited through self-administered questionnaires with anonymity [32].

#### Bribery

Bribery is found to have a negative correlation with health outcomes in Vietnam including enrollment in health insurance programmes [33]. Again, in Vietnam, advice on choice of hospitals from social interactions with friends and relatives has been shown to increase the propensity of bribes to hospital staff and the size of the bribe amount [34]. Bribery has also been found to reduce immunization coverage, delay newborn vaccination, and thus discourage use of public sector health services in the Philippines’ [35]. In Bangladesh, the more remote the location, the greater the size of the bribe [36]. Here, bribery also occurs when recruiting doctors and in decisions regarding posting and transfer to a facility or region of choice [37]. In addition, practices like paying-off management authorities for taking unlawful leave, stealing government revenues such as patient registration fees, taking the salary of a deceased or “ghost” worker, and making false bills for events like training that didn’t occur or in which the incumbent didn’t participate, are common across the region [36, 37].

#### Absenteeism

Reports of widespread absenteeism and negligence were common across the papers in this review. Absences from work without leave were most commonly reported among doctors in the public sector, followed by lab technicians and nurses [28, 38, 39]. In a district in southern India, it was found that around 30% of medical doctors were absent on the day of survey (in 30 selected community health centres) [28]. Only 19% of them were present at all times, although many reported making arrangements with other doctors so that patients were not left unattended. In urban community health centres in Indonesia (9 in a city in Sumatra), 26% of the doctors were absent [38]. In a review of absenteeism in developing countries in 2006, absenteeism was found to vary from 40 to 50% in LMICs of south/south-east countries (India, Bangladesh, Indonesia) [39]. Moreover, regulatory regimes to manage absenteeism are weak and fragmented, and alignment or coordination with laws related to various aspects of health is lacking [40]. Of further concern is the negative impact of absenteeism on the extent to which service seekers trust the health system, and its disproportionate effects on the disadvantaged whose access to health care is already compromised [41].‘Dual practice’ by health professionals is quite common in south/south-east Asia and its poor regulation also encourages absenteeism [42].

#### Underlying causes and consequences of corruption

The underlying causes of irregularities and informal practices emerging from our review were predominantly financial including poor salary and benefits, lack of/poor incentives, lack of autonomy of local authorities to hire and fix remuneration, and lack of accountability of doctors to local authorities [30, 32, 33, 36, 38, 39]. The impacts of corruption are multiple, affecting service access, utilization and cost. Corruption practices associated with the misuse of available resources in resource-constrained settings and increased financial burden on the poorest [30], also lead to inefficient public expenditure [32]. Corruption can increase the cost of treatment to patients if a bribe is demanded or an informal payment is made in addition to the official payment, and thereby reduces demand for services and worsen health outcomes [35]. Other effects include failure to ensure timely and appropriate treatment care for those who can least afford costly services from the private sector [38], and wastage of resources [39].

### Governance

Table 5 summarizes findings from the review of included papers (again, the majority from India and Bangladesh) related to the role of governance in curbing corruption. These are presented below under different sub-titles.

#### Transparency and accountability

Findings reveal that governance failure may occur due to an absence of long-term vision and planning, lack of a functioning regulatory regime, and limited attempts to assess performance of anti-corruption measures. This is especially apparent in the lack of effective accountability mechanisms guiding both private and public health care sectors in Bangladesh [43, 44]. Dissemination of relevant information to the public is lacking which contributes to the public not being properly informed about what services are available, at what cost, and when. For example, in a qualitative study of three districts in Nepal, only 54% of the health facilities were found to have a properly displayed signboard, 47% displayed a citizen charter, and 40% displayed information about free health care services [45]. Moreover, the process of HRH management lacks transparency in LMICs such as India and Indonesia, thereby affecting accountability and trust in the system [46, 47]. In the public sector, about 1/5th of the sanctioned posts in Bangladesh remain vacant, however, the posted doctors seem to be indifferent regarding attendance [48].

#### Citizen’s voice

One way to rectify the weak system of accountability is to adopt demand-side strategies such as increasing the engagement of the citizens and raising their voices as “watch dogs” to claim rights to quality health care services [49, 50]. This may take various forms with varying degrees of effectiveness such as making service providers explain their activities in ‘public hearings’ as has been observed in India [51]. Public hearings as an accountability mechanism have potential to raise critical consciousness among disadvantaged groups such as poor women, and provide a floor in which they can raise their concerns. Better communicating and reconciling provider and citizen expectations is also important, helping improve the Quality of Care [52]. For this process to succeed, fiscal space at the local level is also necessary [53].

#### Decentralisation

In the reviewed literature from Nepal [54] and India [55], decentralization has been advocated as an effective measure to ensure and enhance transparency and accountability at the local level in a manner that addresses community’s needs and priorities. In Nepal, it has been shown to improve access to, utilisation of, and management of health care services [54]. Additionally, suggestions have been made to measure its effects in multiple dimensions while evaluating health outcomes in India [55]. Another qualitative paper from Nepal draws attention to elements such as the roles, rights and responsibilities of both citizens and local institutions in the decentralizing process [56], and how resource constraints at the local level may negatively impact service delivery as has been seen in the case of Indonesia [57].

#### Good governance

Finally, some of the literature sheds light on what constitutes good governance and what needs to be done to achieve it. For example, the use of information technology (IT) is suggested to reduce corruption in public transactions such as procurement of medical supplies as have been observed in India and Bangladesh [58]. On the other hand, insufficient institutional support can undermine people’s participation which is a crucial parameter of good governance. Administrative factors, bureaucratic norms, and motivational patterns are also emphasized as important determinants. In Bangladesh, good governance initiatives are limited and social accountability remains elusive [59]. Achieving good governance for health is the responsibility of citizens as well as political leadership. It requires concerted actions by the justice system and a committed media to mobilise necessary commitments as shown in India [60].

### Regulatory regime

Over the last 12 years, attempts have been made to develop policies to contain corruption in the studied LMICs of the south and south-east Asian region. (Table 6). The six countries in this review signed and adopted the ‘United Nation Convention against Corruption (UNCAC)’ and initiated activities to comply with the Convention [69]. The Right to Information Act (RTI) has also been passed in Bangladesh [61], India [62] and Nepal [63] and Sri Lanka [64], however, implementation remains a challenge. In Bangladesh, for example, the RTI Act has been in place since 2008, yet 29% of citizens continue to face difficulties in accessing information, and 8% reportedly paid bribes to access information [61]. Part of this failure in implementation relates to lack of awareness about the legislation due to insufficient promotion by the government. The Whistle Blower Protection Act has been passed in Bangladesh [65] and India [66] in 2014, however, due to inadequate power of law-enforcing agencies to investigate complaints and impose penalties, implementation has been partial. This is also true for the Ombudsman act [67] and the prevention of corruption act (amendment) to punish bribery [68], both by India.

**Table 6:** Summary of policies related to prevention of corruption in the LMICs of south and south-east Asian countries

Country, year (Ref)	Name of policy	Key feature
Bangladesh, 2008 [61]	Right to Information Act	• Every citizen of Bangladesh shall have the right to information from the authority, and the authority shall, on demand from a citizen, be bound to provide him with the information. • Every authority shall prepare catalogue and index of all information and preserve it in an appropriate manner.
India, 2005 [62]		• An Indian citizen can apply for and obtain information held by any public authority, subject to certain defined exceptions in respect of national interest, legislative privilege and right to privacy.
Nepal, 2007 [63]		• Every citizen of Nepal shall have access to the information held in the public bodies. • **Protection of whistleblower:** It shall be a responsibility of an employee of a public body to provide information on any ongoing or probable corruption or irregularities or any deed taken as offence under the prevailing laws. • The whistleblower shall not be terminated from his/her post or punished with any legal responsibility or caused any loss or harm for giving information.
Sri Lanka, 2016 [64]		• An act to provide for the right of access to information; to specify grounds on which access may be denied; to establish the right to information commission; to appoint information officers; to set out the procedure and for matters connected therewith or incidental thereto.
Bangladesh, 2011 [65]	The Whistle Blower Protection Act	• If any whistleblower discloses any authentic information, his identity cannot not be divulged without his consent. • For making disclosure of public interest information, no criminal or civil, or departmental suit can be filed against the whistleblower. • If the whistleblower is a service holder, only for disclosing public interest information- demotion, harassment transfer or forced retirement or any other measures cannot be taken against him that would incur loss of his psychological, financial or social standing or no departmental actions can be taken against him or he cannot be treated discriminatorily.
India, 2014 [66]		• Establish a mechanism to safeguard persons who make a complaint regarding an act of corruption
India, 2013 [67]	Lokpal and Lokayuktas Act	• Lokpal and Lokayuktas bodies have been empowered to investigate allegations of corruption against public functionaries. • The jurisdiction of the Lokpal (Ombudsman) includes the prime minister, ministers, members of parliament and other public servants.
India, 2013 [68]	Prevention of Corruption (amendment) Bill	• The Amendment Bill include the offense of passive bribery, its various aspects including solicitation and acceptance of bribe through intermediaries (private persons).

## Discussion

This scoping review identified different forms of corruption and its practices among health care providers delivering frontline (PHC) services in selected south/south-east Asian LMICs. Our findings reveal important insights regarding the types, causes, consequences and structural dimensions of such practices as they relate to health outcomes, especially for the poor and disadvantaged. We also reflected on the governance and regulatory arrangements in these countries, and the need for innovative measures for curbing corruption and improving governance, given the largely ineffective traditional (top-down) approaches [70]. The implications of these findings for achieving UHC by 2030 in the context of sustainable development goals (SDGs) are discussed.

The ‘pandemic’ of corruption in frontline health care service delivery is threatening the global goal of achieving UHC by 2030 and health-related SDGs [71]. It is responsible for an estimated loss of over US$500 billion every year, more than what is needed to implement UHC globally. LMICs in the south/south-east Asia region are no exception. Despite its cost to people and health systems in these countries, there exists substantial evidence gaps as shown by the relative scarcity of relevant literature except for India (and to some extent, Bangladesh) which provided the majority of the reviewed papers. This may be due to factors such as the sensitive nature of the topic, a consensus high up in the sector to maintain the ‘dirty secret,’ the ‘political correctness’ of pursuing the issue [72] and last but not the least, the methodological challenges of doing research on corruption [73].

The different forms and dimensions of corruption in south/south-east Asian countries revealed in this study are not new [73, 74]. Whether the corruption is ‘petty’ or not, the cumulative impact on health outcomes is damaging, and affects the equity, quality and responsiveness of the services delivered. Another important consequence of corruption is the erosion of ‘trust’ in the system which reduces utilization and undermines actions to ‘prevent, detect and respond’ to major health crisis such as seen in case of Ebola [75]. Whatever the form and dimension of corruption, a main driver is the ubiquitous financial and structural problem of not offering appropriate incentives (monetary and non-monetary) to motivate the frontline health care providers. Irregularities are further enabled by weak health system characterized by poor governance e.g., poor supervision, victim-blaming, concentration of responsibilities and authorities at the centre, and almost no transparency and accountability [70, 71].

Our review also found substantial gaps in the implementation of the traditional ‘top-down’ measures for containing corruption, resulting in substantial costs for health systems and health service recipients. Some of the existing laws and policies we documented (Table 6) were inconsistent with international standards. One of the reasons may be that the discretion and freedom enjoyed by the frontline providers (as ‘street-level bureaucrats’) [76] in a weak health system encourage them to bend the rules and laws in their favour, and make centrally designed anti-corruption measures largely in-effective. In extreme cases, communities may retaliate by imposing ‘rude accountability’ through social norms and actions that force the providers to deliver services [77].

### What are the alternatives?

The ‘failure’ of traditional measures to contain corruption compels those at high levels of policy and practice to look elsewhere for a new vision which embraces innovative, demand-side, and community perspectives [23]. Whatever the approach adopted, it is essential that health care providers are held accountable to service users, and that systems and providers enable service responsiveness, quality and affordability for better health outcomes [78]. In order to amplify the ‘voice’ of the community, measures are needed that raise awareness about service entitlements and enable complaints to be received and addressed within a reasonable timeframe [79]. It is also imperative that corruption mitigation efforts be comprehensive (multi-pronged and multi-sectoral) and not stand-alone; that they focus on the most harmful practices first and are grounded in grassroots realities; that engage and empower the community and get their buy-in; and be part of a wider health systems reforms to achieve UHC [71].

It is encouraging to note that in a number of countries a variety of innovative bottom-up, and community-based, demand-side interventions are being tested to curb the harmful effects of corruption and make the system more accountable. Examples include: patients’ welfare committees and hospital management societies ensuring responsible use of funds and the provision of quality services in India [80]; primary health care (PHC) management committees that actively engage the community in improving quantity and the quality of services in Nepal [56]; and community monitoring of service provider’s attendance at facilities, especially by women who are the major consumers of the frontline services in Bangladesh [81] and public hearings to voice community’s grievances with health care services and demand remedial measures, again in Bangladesh [82]. However, until and unless these small-scale, local-level experiments are taken to scale, in combination with more effective and efficient traditional regulatory approaches, curbing corruption in the delivery of frontline PHC services will remain elusive.

### Limitations

The search engine used for this scoping study was limited to PubMed, SCOPUS and Google Scholar databases and grey material search to institutional and government websites through Google, due to constraints in time and resources. Only articles and documents in English were searched. We limited our search to the delivery of health care services only and did not include other major areas of health sector corruption such as pharmaceutical procurement and construction of infrastructure. The study would have benefited from perspectives and experiences of the key actors involved at the top levels which was not logistically possible.

### Implications

Interestingly, the countries studied are characterized by low levels of GDP, low levels of education, low levels of democratic values (e.g., freedom of press, speech and congregation etc.) in the political system, and societies organized along strong patriarchal norms. All of these may act as barriers to reduce corruption in these countries [76]. To tackle this situation, a two pronged strategy is recommended: alleviating the socioeconomic, normative and cultural barriers as mentioned above but at the same time, testing community-based, innovative micro-level interventions (such as public hearings, community monitoring of attendance of the health care providers etc.). This will help in the creation of a win-win situation for frontline health care workers as well as vested socio-political interests through improvement of community health and well-being and thereby goodwill for those in power who otherwise benefit from maintaining the *status quo.* Further research is needed to develop and refine such innovations on the ground and to test the feasibility of scaling up the successful ones. Being a sensitive issue, the methods for research on ‘corruption’ also need re-thinking regarding how the key actors of corruption can be challenged and engaged in divulging sensitive information without repercussion.

## Conclusions

Corruption is “embedded” in the health sector [83] and is an open secret [84]. It undermines the development of a pro-poor, equitable and inclusive health system essential for achieving UHC by 2030. This study is first of its kind to document the levels, extents, causes and consequences of corruption in the health sector in the selected LMICs of the south and south-east Asian region. In addition, it explored alternative, bottom-up ways of combating health sector corruption which might guide future activities in this area to improve population health outcomes. Evidently, ‘business as usual’ will not do and given the limitation of traditional measures of containing corruption, strategic ‘out of box’ thinking will be needed. The historic opportunity provided by the SDGs should be seized, and used to push the UHC agenda forward. In the process, it is essential that key players in and around frontline service delivery points are not antagonized, that community is engaged, and that intervention(s) are in alignment with the prevailing political settlements such that efforts towards curbing corruption at the grassroots (e.g., PHC services) are embraced and sustained [85].

## Supplementary information

**Additional file 1.** Preferred Reporting Items for Systematic reviews and Meta-Analyses extension for Scoping Reviews (PRISMA-ScR).

## Data Availability

All data relevant to the study are included in the article. Any additional data may be available from the corresponding author (SMA) on reasonable request.

## Abbreviations

AMR: Antimicrobial Resistance
IT: Information technology
ICTs: Information and Communications Technology
LMICs: Low- and Middle Income-Countries
NGO: Non-GovernmentOrganisation
PRISMA: Preferred Reporting Items for Systematic Reviews and Meta-Analysis
PHC: Primary Health care
RTI: Right to Information
SDGs: Sustainable Development Goals
UHC: Universal Health Coverage
UNCAC: United Nation Convention against Corruption
