# Correction to: The influence of corruption and governance in the delivery of frontline health care services in the public sector: a scoping review of current and future prospects in low and middle-income countries of south and south-east Asia

**DOI:** 10.1186/s12889-020-09197-0

**Published:** 2020-07-09

**Authors:** Nahitun Naher, Roksana Hoque, Muhammad Shaikh Hassan, Dina Balabanova, Alayne M. Adams, Syed Masud Ahmed

**Affiliations:** 1 BRAC James P. Grant BRAC School of Public Health, BRAC University, 5th Floor (Level-6), icddrb Building, 68 ShahidTajuddin Ahmed Sarani, Mohakhali, Dhaka, 1212 Bangladesh; 2 Department of Global Health and Development, London School of Hygiene and Tropical Medicine (LSHTM), Room TP 308, 15-17 Tavistock Place, London, WC1H 9SH UK; 3 Department of Family Medicine, Faculty of Medicine, McGill University, 5858 Cote des Neiges, Room 332, Montréal, Québec, H3S 1Z1 Canada

**Correction to: BMC Public Health (2020) 20:880**

**https://doi.org/10.1186/s12889-020-08975-0**

It was highlighted that the original article [1] contained an error in the Acknowledgment and the Funding section. This Correction article shows the correct Acknowledgment section and Funding section.

**Acknowledgments**

Authors acknowledge support from SamiunNazrinBente Kamal Tune, Senior Research Associate, BRAC James P. Grant BRAC School of Public Health, BRAC University for helping with organizing and formatting the references. This study is part of a large multi-country partnership, the SOAS Anti-Corruption Evidence (ACE) research consortium. For more information on SOAS-ACE visit: www.ace.soas.ac.uk

**Funding**

This publication is an output of the SOAS Anti-Corruption Evidence (ACE) research consortium funded by UK aid from the UK Government [Contract PO 7073]. The views presented are those of the author(s) and do not necessarily reflect the UK government’s official policies or the views of SOAS-ACE or other partner organisations.
